# Impact of cardiac resynchronization therapy in patients with left ventricular assist devices: A systematic review and meta-analysis

**DOI:** 10.1016/j.jhlto.2025.100476

**Published:** 2025-12-30

**Authors:** Miloud Cherbi, Paul Gautier, Raphael Martins, Romain Itier, Laurence Barde, Philippe Maury, Clément Delmas

**Affiliations:** a Cardiology Department, Toulouse University Hospital, Toulouse, France; b Univ Rennes, CHU Rennes, CIC 1414, INSERM, LTSI - UMR 1099, F-35000 Rennes, France

**Keywords:** Left ventricular assist device, Cardiac resynchronization therapy, Ventricular arrhythmia, Ventricular tachycardia, Ventricular fibrillation, Heartmate, Heartware

## Abstract

**Background:**

Many patients with left ventricular assist devices (LVADs) have cardiac resynchronization therapy (CRT). However, the impact of CRT on their clinical and hemodynamic outcomes remains unclear.

**Methods:**

We conducted a systematic review and meta-analysis to evaluate CRT's impact on survival in LVAD patients. We searched PUBMED, EMBASE, and Cochrane databases from inception through April 30, 2025, for studies reporting outcomes in LVAD patients with CRT. The primary outcome was all-cause mortality in patients with versus without CRT. Secondary clinical outcomes included ventricular arrhythmias (VAs) and shocks delivered. Hemodynamic outcomes included heart rate, right atrial pressure, mean pulmonary artery pressure, pulmonary capillary wedge pressure, thermodilution cardiac output, pulmonary artery saturation, right ventricular stroke work index, and left ventricular end-diastolic diameter.

**Results:**

Thirteen studies, including 3,665 patients, were analyzed. Cardiac resynchronization therapy (CRT) did not demonstrate any significant survival benefit, whether comparing CRT-D versus ICD (OR 1.12 [0.85-1.48]), CRT on versus CRT off (OR 1.48 [0.87-2.53]), CRT versus no device (OR 0.99 [0.61-1.59]), or CRT versus no device or ICD (OR 1.00 [0.16-6.31]). Similarly, none of the tested comparisons showed significant differences in VAs incidence or shock rates. Biventricular pacing demonstrated no advantage for any hemodynamic outcomes, whether compared to right ventricular pacing or intrinsic rhythm.

**Conclusion:**

In this meta-analysis, CRT was not associated with overall survival benefit in LVAD recipients, nor with hemodynamic improvement. Future randomized trials may be warranted to definitively establish CRT's value in this population and refine patient selection criteria for optimal outcomes.

## Graphical Abstract

**Figure:**
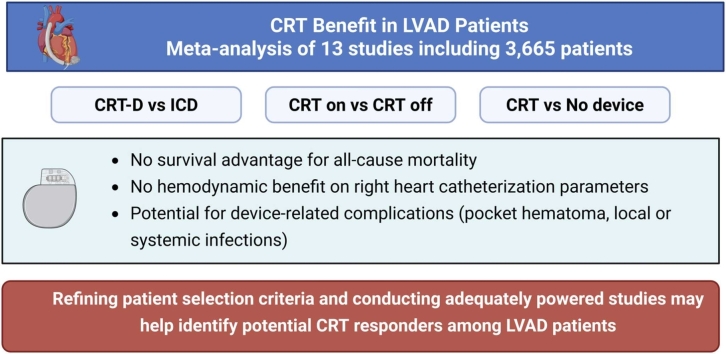

## Background

The growing incidence of advanced heart failure has led to the widespread use of left ventricular assist devices (LVADs) as a cornerstone of management in selected patients. These devices offer significant improvements in both survival and quality of life and are now routinely used as either bridge-to-transplant or destination therapy. Clinical practice guidelines reflect this role, recommending LVAD implantation with strong evidence for patients with INTERMACS profiles 1 to 3, and with more limited endorsement for profile 4.^1^

Despite these therapeutic advances, many aspects of LVAD patient management remain insufficiently defined. Among them is the role of cardiac resynchronization therapy (CRT), which is frequently present at the time of LVAD implantation due to preexisting indications in patients with systolic dysfunction.^2^ While CRT has demonstrated clear benefits in patients with native hearts,^3^ its clinical value in the context of mechanical circulatory support remains uncertain, as observational studies have not consistently shown any survival or symptomatic advantage in LVAD recipients.^4^, ^5^ Furthermore, maintaining CRT in LVAD recipients may expose patients to additional risks, including device-related complications and procedural morbidity—particularly during pulse generator replacements—such as pocket hematomas, pocket infections, and even LVAD-related infections.^6^ In the absence of definitive evidence or standardized guidance, clinical decision-making regarding CRT in LVAD recipients remains highly variable.^2^, ^7^ Most studies conducted to date, however, have not been adequately powered to assess meaningful clinical endpoints such as all-cause mortality or rehospitalization, and their findings may be limited by small sample sizes or the single-center nature of their designs.

Therefore, we conducted this meta-analysis to evaluate the potentials benefits of CRT in LVAD recipients.

## Methods

### Search strategy

Databases (PubMed, Embase, and the Cochrane Central Register of Controlled Trials) were systematically searched, without language restrictions, for all studies—both observational and randomized controlled trials (RCTs)—reporting the outcomes and prognosis of LVAD recipients with CRT, using keywords such as "CRT," and "LVAD" (full strategy in Supplementary Table 1), from inception to April 30, 2025. Searches of unpublished grey literature were performed manually on Google Scholar, OpenGrey, and pre-print servers (MedRxiv). ClinicalTrials.gov was also searched for ongoing studies on CRT and LVAD. The search was kept up to date through automated alerts from PubMed for newly published articles. Institutional review board approval was not required, as this study is a meta-analysis of previously published data. The study was registered in PROSPERO, CRD420251042839. The meta-analysis was performed in line with recommendations from the Cochrane Collaboration and the Preferred Reporting Items for Systematic Reviews and Meta-Analyses Statement (Preferred Reporting Items for Systematic Reviews and Meta-Analyses checklist provided in Supplementary Table 2).8., 9

### Outcomes

The primary outcome was all-cause mortality in LVAD recipients with versus without CRT. Secondary clinical outcomes included the incidence of ventricular arrhythmias (VAs) and the number of shocks delivered by CRT-D or implantable cardioverter-defibrillators (ICDs). Hemodynamic parameters assessed comprised heart rate, right atrial pressure (RAP), mean pulmonary artery pressure (MPAP), pulmonary capillary wedge pressure (PCWP), thermodilution cardiac output (TDCO), pulmonary artery (PA) saturation, right ventricular stroke work index (RVSWI), and left ventricular end-diastolic diameter.

### Article selection

Titles and abstracts of studies retrieved using the search strategy were independently screened by 2 investigators (C.D. and M.C.) to identify studies meeting our inclusion criteria: (1) studies reporting outcomes of patients after their first LVAD implantation; and (2) reporting rates of at least one of the clinical or hemodynamic outcomes of interest mentioned above. Conference abstracts and case reports were not included. The full texts of potentially eligible studies were retrieved and independently assessed for eligibility by the 2 investigators. Discrepancies were resolved through discussion, and final decisions were made by consensus.

### Data extraction

Two authors (C.D. and M.C.) independently extracted data on study characteristics (including study ID, design, protocol, sample size, and follow-up period), baseline patient characteristics (such as sex, age, ejection fraction, ischemic cardiomyopathy, and history of VA), and all clinical and hemodynamic outcomes.

### Risk of bias, quality assessment, and certainty of evidence

Given the limited number of studies per comparison (<10), formal statistical tests for publication bias (Egger's test, funnel plots) were not performed, as they lack sufficient power to detect bias reliably.8., 9 Instead, we conducted a comprehensive search strategy, including grey literature and trial registries to minimize publication bias. For observational studies, quality was assessed using the ROBINS-I tool,^10^ which considers 7 domains: confounding factors, classification of interventions, selection of participants, deviations from intended interventions, missing data, measurement of outcomes, and selection of the reported results. For RCTs, quality assessment was performed using the Cochrane Risk of Bias (RoB2) tool for crossover trials.^11^ Two authors (C.D. and M.C.) independently assessed the quality of the studies, using the Grades of Recommendation, Assessment, Development and Evaluation (GRADE) approach to assess the quality of evidence for each outcome.^12^ Disagreements between reviewers were resolved by discussion.

### Statistical analysis

For dichotomous outcomes, the number of events and total sample size in each group were extracted from each study for each outcome of interest. A pooled analysis was then conducted by aggregating the data to calculate the odds ratios (ORs) and corresponding 95% confidence intervals (CIs) for each dichotomous outcome. For quantitative outcomes, standardized mean differences (SMDs) and their standard errors (SEs) were calculated using Cohen’s d. For studies reporting raw data (means, standard deviations, and sample sizes), SMDs and their corresponding SEs were computed using standard formulas.^13^ For studies providing pre-calculated SMDs with standard deviations of the difference, the standard deviations were converted to SEs using SE = SD/√n, where n represents the total sample size. The DerSimonian and Laird random‐effects model was employed because, based on preexisting clinical and pathophysiological considerations,^2^, ^4^, ^5^, ^7^ we could not rule out the possibility that the observed estimates of CRT effect might vary across studies because of real differences in the CRT effect in each study as well as sampling variability.8., 14 Between-study heterogeneity was estimated using the restricted maximum likelihood method. The Hartung-Knapp-Sidik-Jonkman correction was applied to derive more robust CIs.^8^ Statistical heterogeneity was initially assessed using the I² statistic, which represents the proportion of total variation observed between trials that is attributable to differences between trials rather than sampling error (chance), with I² ≥ 50% considered indicative of substantial heterogeneity.^8^ Analyses were performed using R software (version 4.3.2) with the *meta* and *metafor* packages.

## Results

### Baseline characteristics

**Figure 1:**
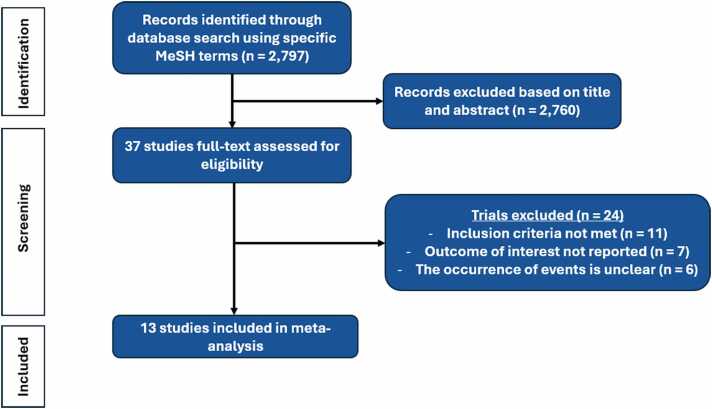
Flow chart of the study.

From 2,797 references retrieved through the search strategy, 37 studies were selected for full-text assessment of inclusion criteria based on title and abstract screening. Flow chart of literature retrieval and reasons for article exclusion are shown in Figure 1. Finally, 13 studies met inclusion criteria, that included a total of 3,665 patients. Among them, 2 were RCTs (45 patients), and 4 were multicentric studies (2,694 patients). For clinical outcomes, 4 comparisons were possible based on available data: (1) patients with CRT-D versus ICD (4 studies, 2,141 patients); (2) patients with CRT in whom biventricular pacing was turned off after LVAD implantation (CRT on vs CRT off, 3 studies, 640 patients); (3) patients with CRT versus those with no device (2 studies, 973 patients); and (4) patients with CRT versus those with either ICD or no device (2 studies, 1,911 patients). For hemodynamic outcomes, 3 comparisons were feasible: (1) biventricular pacing versus right ventricular (RV) pacing (4 studies, 74 patients); (2) biventricular pacing versus intrinsic rhythm (2 studies, 37 patients); and (3) CRT on versus CRT off (2 studies, 327 patients). The main characteristics of the studied populations are presented in Table 1.

**Table 1:** Summary of Main Characteristics of Studies Included in the Meta-Analysis.

	Design	Country	Number of patients	Available comparisons	Outcomes included in the meta-analysis	Mean/median age, years	Male sex, n (%)	LVAD device, n (%)
Gopinathannair et al, 2018^15^	Observational Retrospective Multicenter	USA	488 patients CRT-D: 265 patients ICD: 223 patients	CRT-D vs ICD	– All-cause mortality – Ventricular arrythmias – Shocks	57.9 years CRT-D: 60.4 ICD: 55.0	395 (80.9)	HM2: 410 (84.0) HVAD: 78 (16.0)
Joly et al, 2018^16^	Observational Prospective Single center	USA	7 patients	Biventricular pacing vs RV pacing Biventricular pacing vs intrinsic rhythm	– RAP – MPAP – PCWP	50.4 years	Not specified	Not specified
Tehrani et al, 2019^17^	Observational Prospective Single center	USA	62 patients Active CRT pacing: 25 patients Non-active CRT pacing: 37 patients	Active CRT pacing vs non-active CRT pacing	– RAP – MPAP – PCWP – LVEDD	59.6 years Active CRT: 63.8 Non-active CRT: 56.8	37 (59.7)	HM2: 42 (67.7) HVAD: 20 (32.3)
Cotarlan et al, 2019^18^	Observational Prospective Single center	USA	22 patients	Biventricular pacing vs RV pacing Biventricular pacing vs intrinsic rhythm	– HR – RAP – MPAP – PCWP – TDCO – PA saturation – RVSWI	62.0 years	21 (95.5%)	HM2: 20 (90.9%) HM3: 2 (9.1%)
Roukoz et al, 2020^19^	Observational Retrospective Multicenter	USA	295 patients CRT-on: 251 patients CRT-off: 44 patients	CRT-on vs CRT-off	– All-cause mortality – Ventricular arrythmias – LVEDD	60.5 years CRT-on: 60.0 CRT-off: 63.0	285 (83.0)	HM2: 253 (85.8) HVAD: 42 (14.2)
Chung et al, 2021^20^	Randomized trial Crossover Single center	USA	30 patients	Biventricular pacing vs RV pacing	– HR	65 years	21 (70.0)	HM2: 15 (50.0) HM3: 10 (33.3) HVAD: 5 (16.7)
Darden et al, 2021^21^	Observational Retrospective Multicenter	USA, Italy, Croatia, Sweden, Belgium, Netherlands	524 patients No device: 146 patients ICD: 239 patients CRT-P: 28 patients CRT-D: 111 patients	CRT vs no-CRT CRT vs no-device CRT-D vs ICD	– All-cause mortality	52.4 years No device: 49.9 ICD: 52.6 CRT-P: 52.6 CRT-D: 55.1	442 (84.4)	HM2: 314 (59.9) HVAD: 210 (40.1)
Tomashitis et al, 2021^22^	Randomized trial Crossover Single center	USA	15 patients	Biventricular pacing vs RV pacing Biventricular pacing vs intrinsic rhythm	– HR – RAP – MPAP – PCWP – TDCO – PA saturation – RVSWI	58.0 years	10 (66.7)	HM2: 6 (40.0) HM3: 6 (40.0) HVAD: 3 (20.0)
Chou et al, 2022^23^	Observational Retrospective Single center	USA	186 patients CRT-on: 63 patients CRT-off: 123 patients	CRT-on vs CRT-off	– All-cause mortality – Ventricular arrythmias	52.8 years CRT-on: 59.7 CRT-off: 49.2	139 (74.7)	HM2: 3 (1.6) HM3: 111 (59.7) HVAD: 70 (37.6)
Gulletta et al, 2022^24^	Observational Retrospective Single center	Italy	69 patients Active CRT pacing: 27 patients Non-active CRT pacing: 42 patients	Active CRT pacing vs non-active CRT pacing	– All-cause mortality – Ventricular arrythmias	66.0 years	65 (94.2)	HM2: 1 (1.4) HM3: 33 (47.8) HVAD: 35 (50.7)
Andreae et al, 2025^25^	Observational Retrospective Single center	USA	421 patients CRT-D: 185 patients ICD: 236 patients	CRT-D vs ICD	– Shocks	63.0 years	342 (81.2)	HM2: 172 (40.9) HM3: 129 (30.6) HVAD: 120 (28.5)
Oates et al, 2025^5^	Observational Retrospective Single center	USA	159 patients CRT-on: 74 CRT-off: 85	CRT-on vs CRT-off	– All-cause mortality – Ventricular arrythmias	62.9 years CRT-on: 68.5 CRT-off: 66.1	122 (76.7)	HM2: 38 (23.8) HM3: 66 (65.8) HVAD: 55 (34.6)
Sayer et al, 2025^4^	Observational Prospective Multicenter	USA	1,387 patients No device: 153 patients ICD: 699 patients CRT-D: 535 patients	CRT-D vs no-CRT CRT-D vs no-device CRT-D vs ICD	– All-cause mortality – Ventricular arrythmias	59.6 years No device: 55.8 ICD/CRT-D: 60.1	1,098 (79.2)	HM3: 1,387 (100.0)

CRT, cardiac resynchronization therapy; HM2, Heartmate 2; HM3, Heartmate 3; HR, heart rate; HVAD, Heartware; ICD, implantable cardioverter-defibrillator; LVAD, left ventricular assist device; LVEDD, left ventricular end-diastolic diameter; MPAP, mean pulmonary artery pressure; PA, pulmonary artery; PCWP, pulmonary capillary wedge pressure; RAP, right atrial pressure; RV, right ventricular; RVSWI, right ventricular stroke work index; TDCO, thermodilution cardiac output.

### Clinical outcomes

**Figure 2:**
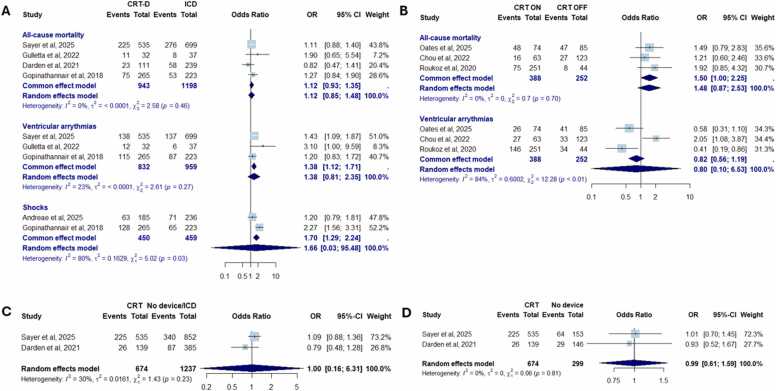
Meta-analysis of clinical outcomes assessing the impact of CRT in LVAD recipients. Panel A shows the comparison between CRT-D and ICD. Panel B presents the comparison between CRT on and CRT off. Panel C illustrates the comparison between CRT and no device or ICD. Panel D shows the comparison between CRT and no device. CI, confidence interval; CRT, cardiac resynchronization therapy; ICD, implantable cardioverter-defibrillator; LVAD, left ventricular assist device; OR, odds ratio.

In the first analysis comparing patients with CRT-D versus ICD, no significant differences were observed between groups for any clinical outcomes, including all-cause mortality (OR 1.12 [0.85-1.48]; low heterogeneity, I² = 0%) and incidence of VAs (OR 1.38 [0.81-2.35]; low heterogeneity, I² = 23%) (Figure 2A). Moreover, there was no significant difference in the rate of delivered shocks between CRT-D and ICD (14.8 vs 9.4 per 100 person-years, OR 1.66 [0.03-95.48]; high heterogeneity, I² = 80%). Similarly, in the comparison between CRT on and CRT off, no significant differences were found in all-cause mortality (OR 1.48 [0.87-2.53]; I² = 0%) or incidence of VAs (OR 0.80 [0.10-6.53]; high heterogeneity, I² = 84%) (Figure 2B). Finally, no significant differences in all-cause mortality were observed between patients with CRT and those with either no device or an ICD (OR 1.00 [0.16-6.31]; I² = 30%, Figure 2C), or between patients with CRT and those without any device (OR 0.99 [0.61-1.59]; I² = 0%, Figure 2D).

### Hemodynamic outcomes

**Figure 3:**
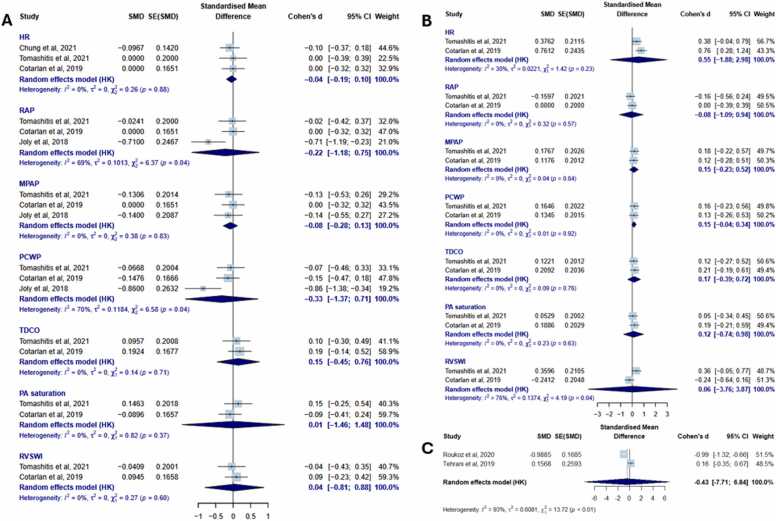
Meta-analysis of hemodynamic outcomes assessing the impact of CRT in LVAD recipients. Panel A shows the comparison between biventricular and RV pacing. Panel B presents the comparison between biventricular pacing and intrinsic rhythm. Panel C illustrates the comparison between CRT on and CRT off for LVEDD. CI, confidence interval; CRT, cardiac resynchronization therapy; HR, heart rate; LVAD, left ventricular assist device; LVEDD, left ventricular end-diastolic diameter; MPAP, mean pulmonary artery pressure; OR, odds ratio; PA, pulmonary artery; PCWP, pulmonary capillary wedge pressure; RAP, right atrial pressure; RV, right ventricular; RVSWI, right ventricular stroke work index; TDCO, thermodilution cardiac output.

In the first comparison between biventricular pacing and RV pacing, no significant differences were observed across all hemodynamic parameters tested, including heart rate (SMD −0.04 [−0.19 to 0.10], I² = 0%), RAP (SMD −0.22 [−1.18 to 0.75], I² = 69%), MPAP (SMD −0.08 [−0.28 to 0.13], I² = 0%), PCWP (SMD −0.33 [−1.37 to 0.71], I² = 70%), TDCO (SMD 0.15 [−0.45 to 0.76], I² = 0%), PA saturation (SMD 0.01 [−1.46 to 1.48], I² = 0%), and RVSWI (SMD 0.04 [−0.81 to 0.88], I² = 0%) (Figure 3A). Similarly, no differences in any of these hemodynamic parameters were found when comparing biventricular pacing to intrinsic rhythm (Figure 3B). Finally, no difference was observed for left ventricular end-diastolic diameter between CRT on and CRT off (SMD –0.43 [–7.71 to 6.84], I² = 93%) (Figure 3C).

### Quality assessment and GRADE framework results

Among the studies included, 6 were assessed as having a low RoB2 and 7 as high RoB2. A detailed quality assessment for each study is presented in Supplementary Table 3. The certainty of evidence for all outcomes across all comparisons was rated as low or very low according to the GRADE system, primarily due to the non-randomized design of the included studies, risk of confounding, limited number of studies, and/or imprecise effect estimates. Further details and justifications are provided in Supplementary Table 4.

## Discussion

To date, we present the largest meta-analysis evaluating the impact of CRT on clinical outcomes and hemodynamic parameters in LVAD recipients, including a total of 3,665 patients. Our main findings are as follows: (1) CRT was not associated with an overall survival benefit in LVAD patients, nor with an increased incidence of VAs, whether compared to patients without CRT or with ICD; (2) Biventricular pacing was not associated with any advantage for any hemodynamic parameters, including RAP, MPAP, PCWP, TDCO, PA saturation, or RVSWI, whether compared to RV pacing or intrinsic rhythm; and (3) despite CRT being frequently pre-existing in LVAD recipients, very few prospective studies have assessed its impact, and further research is needed to clarify its exact role.

With the increasing burden of advanced heart failure and persistent limitations in organ availability,^26^ the use of LVADs is anticipated to grow substantially, making the optimization of care for these patients a key therapeutic objective. While many individuals undergoing LVAD implantation have previously been treated with CRT, the most effective approach to managing CRT in the post-implantation setting remains to be defined.^2^, ^7^ Our findings reinforce the current lack of robust clinical evidence supporting a survival benefit from maintaining CRT after LVAD implantation. Despite some heterogeneity across studies, there was also no indication of an increased risk of VAs. Notably, all assessed hemodynamic parameters—including pressure and flow measurements impacting both the left and right ventricles—remained unchanged with the use of biventricular pacing compared to either right ventricular pacing or intrinsic rhythm.

The lack of clinical or physiological benefit observed with CRT in patients supported by LVADs highlights the need for further focused research. One possible explanation is that the continuous unloading of the left ventricle by the LVAD may diminish the relevance of achieving electrical synchrony or optimizing ventricular loading conditions—mechanisms through which CRT typically exerts its effects. This "LVAD dominance" hypothesis is particularly relevant for right ventricular function, where CRT has been shown to improve hemodynamics in native hearts, especially in patients with elevated right-sided pressures.^3^, ^27^ However, in our meta-analysis, no significant improvement was observed in any RV hemodynamic parameter (RAP, RVSWI) with biventricular pacing, suggesting that the profound hemodynamic effects of mechanical unloading may supersede any potential benefit from electrical resynchronization. Furthermore, in the presence of continuous mechanical support, electrical dyssynchrony may become physiologically silent—that is, while ventricular dyssynchrony persists electrically, its mechanical and hemodynamic consequences are masked by the LVAD's continuous flow output, rendering CRT's corrective effect clinically irrelevant. In addition, there is a theoretical concern that biventricular pacing could unintentionally disrupt ventricular mechanics, potentially displacing the septum or lateral wall toward the inflow cannula, which might predispose to suction-related complications, RV dysfunction, or arrhythmic events. The altered hemodynamic environment created by continuous-flow devices—marked by diminished pulsatility and disturbed vascular signaling—may further reduce the physiological effectiveness of CRT. It is conceivable, however, that CRT may offer selective advantages in specific clinical scenarios, depending on individual cardiac mechanics or structural characteristics. Further research is warranted to clarify these contexts and refine patient selection criteria in order to guide the appropriate use of CRT in the setting of mechanical circulatory support.

In real-world settings, the lack of observed clinical or hemodynamic benefit from continued CRT following LVAD implantation may have practical implications for long-term device strategy. Indeed, biventricular pacing is known to accelerate battery depletion, and procedures associated with generator replacement can be particularly risky in this population, with a higher incidence of complications such as pocket hematomas, infections, and even LVAD-related infections.^6^, ^27^ For patients with CRT-P who maintain an intrinsic rhythm, deferring generator exchange may be a reasonable approach to minimize procedural hazards—especially in light of our findings showing no added hemodynamic benefit from biventricular pacing over intrinsic conduction. Similarly, for pacing-dependent individuals, a transition to RV-only pacing could be considered, as it may preserve battery life without negatively impacting hemodynamic performance, particularly given our findings showing no hemodynamic advantage of biventricular over RV pacing. In patients with CRT-D systems, downgrading to ICD-only therapy while abandoning the LV lead may offer a simpler, lower-risk, and more economical alternative. Finally, emerging conduction system pacing modalities, such as left bundle branch area pacing, represent a promising area for future investigation.^28^ By more closely mimicking physiological activation with a single depolarization wavefront, this strategy could offer distinct effects in the LVAD setting and merits further study.

Overall, these findings emphasize the need for improved methods to identify patients unlikely to respond to CRT prior to device implantation. Those progressing to LVAD support after receiving CRT probably derived limited benefit from resynchronization therapy. Early recognition of such non-responders could prevent unnecessary CRT use, streamline treatment pathways, and enable timely access to advanced therapies like LVAD implantation—or potentially guide CRT removal at the time of LVAD surgery. Considering the lack of proven survival benefit and the associated procedural risks, well-designed RCTs are crucial to establish the true value and patient selection criteria for CRT in the setting of LVAD support.

## Limitations

This meta-analysis is mainly limited by the nature of the available data, which predominantly originate from retrospective, single-center studies, thus exposing the results to potential information bias. Additionally, HeartMate 3 devices were underrepresented among the included patients, which restricts the generalizability of our findings to the current LVAD population. Indeed, HeartMate 3 is now the sole device used for new implantations in clinical practice, although HeartMate 2 and HeartWare still account for a significant proportion of previously implanted devices. Additionally, hemodynamic outcomes were derived from a limited number of studies, several with small sample sizes, and should therefore be interpreted as exploratory findings requiring validation in larger, adequately powered studies. However, these small studies did not contribute to our primary endpoint of all-cause mortality, which demonstrated consistent results with low heterogeneity across larger cohorts*.* Another important limitation is the absence of device-related complication data across the included studies, which should be included in dedicated studies for a comprehensive benefit-risk assessment. Furthermore, despite being the largest meta-analysis on this subject to date, the overall quality of evidence remains modest, and no definitive causal inferences can be drawn without data from large RCTs.

## Conclusion

In this large meta-analysis, CRT was not associated with an overall survival benefit in LVAD recipients, nor with any hemodynamic improvement. Future RCTs may be warranted to definitively establish the role of CRT in LVAD recipients and to refine patient selection criteria for optimal outcomes.

## Data availability statement

The authors declare that all supporting data are available within the article [and its online supplementary files].

## Conflicts of Interest statement

The authors declare that they have no known competing financial interests or personal relationships that could have appeared to influence the work reported in this paper.

## Footnotes

Appendix A Supplementary data associated with this article can be found in the online version at doi:10.1016/j.jhlto.2025.100476.
