# Supplementary material for: Impact of cardiac resynchronization therapy in patients with left ventricular assist devices: A systematic review and meta-analysis
Source: JHLT Open. 2025 Dec 30;11:100476. doi: 10.1016/j.jhlto.2025.100476 (PMC12857394; doi:10.1016/j.jhlto.2025.100476)
Supplement: Supplementary file 1 — Supplementary material [file mmc1.docx]

| PUBMED | ("CRT"[All Fields] OR ("cardiac resynchronization therapy"[MeSH Terms] OR ("cardiac"[All Fields] AND "resynchronization"[All Fields] AND "therapy"[All Fields]) OR "cardiac resynchronization therapy"[All Fields])) AND (("left"[All Fields] AND ("heart assist devices"[MeSH Terms] OR ("heart assist"[All Fields] AND "devices"[All Fields]) OR "heart assist devices"[All Fields] OR ("ventricular"[All Fields] AND "assist"[All Fields] AND "device"[All Fields]) OR "ventricular assist device"[All Fields])) OR "LVAD"[All Fields] OR ("heart assist devices"[MeSH Terms] OR ("heart assist"[All Fields] AND "devices"[All Fields]) OR "heart assist devices"[All Fields] OR ("ventricular"[All Fields] AND "assist"[All Fields] AND "device"[All Fields]) OR "ventricular assist device"[All Fields]) OR "VAD"[All Fields] OR "Heartmate"[All Fields] OR "Heartware"[All Fields] OR "HVAD"[All Fields]) |
| --- | --- |
| Embase | (crt OR 'cardiac resynchronization therapy'/exp OR 'cardiac resynchronization therapy' OR (('cardiac'/exp OR cardiac) AND resynchronization AND ('therapy'/exp OR therapy))) AND ('left ventricular assist device'/exp OR 'left ventricular assist device' OR (left AND ventricular AND assist AND ('device'/exp OR device)) OR 'lvad'/exp OR lvad OR 'ventricular assist device'/exp OR 'ventricular assist device' OR (ventricular AND assist AND ('device'/exp OR device)) OR vad OR 'heartmate'/exp OR heartmate OR 'heartware'/exp OR heartware OR 'hvad'/exp OR hvad) |
| Cochrane | #1 CRT[tiab] OR "cardiac resynchronization therapy"[tiab] OR "Cardiac Resynchronization Therapy"[Mesh]  #2 "left ventricular assist device"[tiab] OR LVAD[tiab] OR "ventricular assist device"[tiab] OR VAD[tiab] OR HeartMate[tiab] OR HeartWare[tiab] OR HVAD[tiab] OR "Ventricular Assist Devices"[Mesh]  #3 #1 AND #2 |

Supplementary Table 1. Details of MeSH Search Terms.

| **Section and Topic** | **Item #** | **Checklist item** | **Location where item is reported** |
| --- | --- | --- | --- |
| **TITLE** | | |  |
| Title | 1 | Identify the report as a systematic review. | Page 1 |
| **ABSTRACT** | | |  |
| Abstract | 2 | See the PRISMA 2020 for Abstracts checklist. | Page 5 |
| **INTRODUCTION** | | |  |
| Rationale | 3 | Describe the rationale for the review in the context of existing knowledge. | Page 4 |
| Objectives | 4 | Provide an explicit statement of the objective(s) or question(s) the review addresses. | Page 4 |
| **METHODS** | | |  |
| Eligibility criteria | 5 | Specify the inclusion and exclusion criteria for the review and how studies were grouped for the syntheses. | Page 5 |
| Information sources | 6 | Specify all databases, registers, websites, organisations, reference lists and other sources searched or consulted to identify studies. Specify the date when each source was last searched or consulted. | Page 5 |
| Search strategy | 7 | Present the full search strategies for all databases, registers and websites, including any filters and limits used. | Pages 5 and 6 |
| Selection process | 8 | Specify the methods used to decide whether a study met the inclusion criteria of the review, including how many reviewers screened each record and each report retrieved, whether they worked independently, and if applicable, details of automation tools used in the process. | Pages 5 and 6 |
| Data collection process | 9 | Specify the methods used to collect data from reports, including how many reviewers collected data from each report, whether they worked independently, any processes for obtaining or confirming data from study investigators, and if applicable, details of automation tools used in the process. | Pages 5 and 6 |
| Data items | 10a | List and define all outcomes for which data were sought. Specify whether all results that were compatible with each outcome domain in each study were sought (e.g. for all measures, time points, analyses), and if not, the methods used to decide which results to collect. | Pages 5 and 6 |
|  | 10b | List and define all other variables for which data were sought (e.g. participant and intervention characteristics, funding sources). Describe any assumptions made about any missing or unclear information. | Pages 5 and 6 |
| Study risk of bias assessment | 11 | Specify the methods used to assess risk of bias in the included studies, including details of the tool(s) used, how many reviewers assessed each study and whether they worked independently, and if applicable, details of automation tools used in the process. | Pages 6 and 7 |
| Effect measures | 12 | Specify for each outcome the effect measure(s) (e.g. risk ratio, mean difference) used in the synthesis or presentation of results. | Pages 7 and 8 |
| Synthesis methods | 13a | Describe the processes used to decide which studies were eligible for each synthesis (e.g. tabulating the study intervention characteristics and comparing against the planned groups for each synthesis (item #5)). | Pages 5-8 and Sup Table 3 |
|  | 13b | Describe any methods required to prepare the data for presentation or synthesis, such as handling of missing summary statistics, or data conversions. | Pages 7 and Sup Table 3 |
|  | 13c | Describe any methods used to tabulate or visually display results of individual studies and syntheses. | Pages 7 and 8 |
|  | 13d | Describe any methods used to synthesize results and provide a rationale for the choice(s). If meta-analysis was performed, describe the model(s), method(s) to identify the presence and extent of statistical heterogeneity, and software package(s) used. | Pages 7 and 8 |
|  | 13e | Describe any methods used to explore possible causes of heterogeneity among study results (e.g. subgroup analysis, meta-regression). | Pages 7 and 8 |
|  | 13f | Describe any sensitivity analyses conducted to assess robustness of the synthesized results. | NA |
| Reporting bias assessment | 14 | Describe any methods used to assess risk of bias due to missing results in a synthesis (arising from reporting biases). | Page 7 |
| Certainty assessment | 15 | Describe any methods used to assess certainty (or confidence) in the body of evidence for an outcome. | Pages 6 and 7 |
| **RESULTS** | | |  |
| Study selection | 16a | Describe the results of the search and selection process, from the number of records identified in the search to the number of studies included in the review, ideally using a flow diagram. | Page 8 and Figure 1 |
|  | 16b | Cite studies that might appear to meet the inclusion criteria, but which were excluded, and explain why they were excluded. | Page 8 and Figure 1 |
| Study characteristics | 17 | Cite each included study and present its characteristics. | Sup Table 3 |
| Risk of bias in studies | 18 | Present assessments of risk of bias for each included study. | Page 9 and Sup Table 4 |
| Results of individual studies | 19 | For all outcomes, present, for each study: (a) summary statistics for each group (where appropriate) and (b) an effect estimate and its precision (e.g. confidence/credible interval), ideally using structured tables or plots. | Figures 2-3 |
| Results of syntheses | 20a | For each synthesis, briefly summarise the characteristics and risk of bias among contributing studies. | Page 9  Sup Table 4 |
|  | 20b | Present results of all statistical syntheses conducted. If meta-analysis was done, present for each the summary estimate and its precision (e.g. confidence/credible interval) and measures of statistical heterogeneity. If comparing groups, describe the direction of the effect. | Figures 2-3 |
|  | 20c | Present results of all investigations of possible causes of heterogeneity among study results. | Figures 2-3 |
|  | 20d | Present results of all sensitivity analyses conducted to assess the robustness of the synthesized results. | NA |
| Reporting biases | 21 | Present assessments of risk of bias due to missing results (arising from reporting biases) for each synthesis assessed. | Sup Table 4 |
| Certainty of evidence | 22 | Present assessments of certainty (or confidence) in the body of evidence for each outcome assessed. | Sup Table 5 |
| **DISCUSSION** | | |  |
| Discussion | 23a | Provide a general interpretation of the results in the context of other evidence. | Pages 10 and 11 |
|  | 23b | Discuss any limitations of the evidence included in the review. | Page 12 |
|  | 23c | Discuss any limitations of the review processes used. | Page 12 |
|  | 23d | Discuss implications of the results for practice, policy, and future research. | Pages 11 and 13 |
| **OTHER INFORMATION** | | |  |
| Registration and protocol | 24a | Provide registration information for the review, including register name and registration number, or state that the review was not registered. | Page 5 |
|  | 24b | Indicate where the review protocol can be accessed, or state that a protocol was not prepared. | Page 5 |
|  | 24c | Describe and explain any amendments to information provided at registration or in the protocol. | NA |
| Support | 25 | Describe sources of financial or non-financial support for the review, and the role of the funders or sponsors in the review. | Page 13 |
| Competing interests | 26 | Declare any competing interests of review authors. | Page 13 |
| Availability of data, code and other materials | 27 | Report which of the following are publicly available and where they can be found: template data collection forms; data extracted from included studies; data used for all analyses; analytic code; any other materials used in the review. | Page 13 |

*From:*  Page MJ, McKenzie JE, Bossuyt PM, Boutron I, Hoffmann TC, Mulrow CD, et al. The PRISMA 2020 statement: an updated guideline for reporting systematic reviews. BMJ 2021;372:n71. doi: 10.1136/bmj.n71

Supplementary Table 2. PRISMA Check list

| **Risk of bias assessment for observational studies (ROBINS-I)** | | | | | | | |
| --- | --- | --- | --- | --- | --- | --- | --- |
|  | Confounding factors | Classification of interventions | Missing data | | Measurement of outcomes | Selection of the reported results | Overall risk of bias |
| Gopinathannair et al, 2018^1^ | High | Low | Low | | Low | Low | High |
| Joly et al, 2018^2^ | Low | Low | Low | | Low | Low | Low |
| Tehrani et al, 2019^3^ | High | Low | Low | | Low | Low | High |
| Cotarlan et al, 2019^4^ | Low | Low | Low | | Low | Low | Low |
| Roukoz et al, 2020^5^ | High | Low | Low | | Low | Low | Low |
| Darden et al, 2021^7^ | High | Low | Low | | Low | Low | High |
| Chou et al, 2022^9^ | High | Low | Low | | Low | Low | High |
| Gulletta et al, 2022^10^ | High | Low | Low | | Low | Low | High |
| Andreae et al, 2025^11^ | High | Low | Low | | Low | Low | High |
| Oates et al, 2025^12^ | High | Low | Low | | Low | Low | High |
| Sayer et al, 2025^13^ | Low | Low | Low | | Low | Low | Low |
| **Risk of bias assessment for randomised studies (RoB2 for crossover trials)** | | | | | | | |
|  | Randomisation process | Carry-over effect | Deviations from  intended interventions | Missing outcome data | Measurement of the outcome | Selection of reported results | Overall risk of bias |
| Chung et al, 2021^6^ | Low | Low | Low | Low | Low | Low | Low |
| Tomashitis et al, 2021^8^ | Low | Low | Low | Low | Low | Low | Low |

Supplementary Table 3. Risk of bias assessment.

**References**

1. Gopinathannair R, Roukoz H, Bhan A, et al. Cardiac Resynchronization Therapy and Clinical Outcomes in Continuous Flow Left Ventricular Assist Device Recipients. *J Am Heart Assoc*. 2018 Jun 15;7(:e009091.
2. Joly JM, Acharya D, Doppalapudi H, et al Acute Hemodynamic Effects of Biventricular Pacing After Left Ventricular Assist Device. *J Card Fail*. 2018 Oct;24:716-718.
3. Tehrani DM, Adatya S, Grinstein J, et al. Impact of Cardiac Resynchronization Therapy on Left Ventricular Unloading in Patients with Implanted Left Ventricular Assist Devices. *ASAIO J.* 2019 Feb;65:117-122
4. Cotarlan V, Johnson F, Goerbig-Campbell J, et al. Usefulness of Cardiac Resynchronization Therapy in Patients With Continuous Flow Left Ventricular Assist Devices. *Am J Cardiol*. 2019 Jan 1;123:93-99.
5. Roukoz H, Bhan A, Ravichandran A, et al. Continued versus Suspended Cardiac Resynchronization Therapy after Left Ventricular Assist Device Implantation. *Sci Rep*. 2020 Feb 13;10:2573.
6. Chung BB, Grinstein JS, Imamura T, et al. Biventricular Pacing Versus Right Ventricular Pacing in Patients Supported With LVAD. *JACC Clin Electrophysiol*. 2021 Aug;7:1003-1009
7. Darden D, Ammirati E, Brambatti M, et al. Cardiovascular implantable electronic device therapy in patients with left ventricular assist devices: insights from TRAViATA. *Int J Cardiol*. 2021 Oct 1;340:26-33.
8. Tomashitis B, Baicu CF, Butschek RA, et al. Acute Hemodynamic Effects of Cardiac Resynchronization Therapy Versus Alternative Pacing Strategies in Patients With Left Ventricular Assist Devices. *J Am Heart Assoc*. 2021 Mar 16;10:e018127.
9. Chou A, Larson J, Deshmukh A, et al. Association between biventricular pacing and incidence of ventricular arrhythmias in the early post-operative period after left ventricular assist device implantation. *J Cardiovasc Electrophysiol*. 2022 May;33:1024-1031.
10. Gulletta S, Scandroglio AM, Pannone L, et al. Clinical characteristics and outcomes of patients with ventricular arrhythmias after continuous-flow left ventricular assist device implant. *Artif Organs*. 2022 Aug;46:1608-1615.
11. Andreae A, Black-Maier E, Arps K, et al. Appropriate and inappropriate ICD shocks in patients with LVADs: Prevalence, associated factors, and etiologies. *Heart Rhythm*. 2025 Feb;22:394-402.
12. Oates CP, Lawrence LL, Bigham GE, et al. Impact of Cardiac Resynchronization Therapy on Ventricular Arrhythmias and Survival After Durable Left Ventricular Assist Device Implantation. *ASAIO J*. 2025 Feb 1;71:157-163.
13. Sayer G, Ahmed MM, Mehra MR, et al. Implantable Cardioverter-Defibrillators and Cardiovascular Resynchronization Therapy with Left Ventricular Assist DevicesA MOMENTUM 3 Trial Analysis. *J Card Fail*. 2025 Jan 22:S1071-916400012-0.

| **Certainty assessment** | | | | | | |
| --- | --- | --- | --- | --- | --- | --- |
| Participants | Risk of bias | Inconsistency | Indirectness | Imprecision | Publication bias | Overall certainty of evidence |
|  |  |  |  |  |  |  |
| **CRT-D vs ICD** | | | | | | |
| All-cause mortality  2,141 patients  4 cohort studies | Serious^a^ | Not serious | Not serious | Not serious | Not serious | ◯⨁◯◯ Low |
| Ventricular arrhythmias  1,791 patients  3 cohort studies | Serious^a^ | Not serious | Not serious | Not serious | Not serious | ◯⨁◯◯ Low |
| Shocks  909 patients  2 cohort studies | Serious^a^ | Not serious | Not serious | Serious^b^ | Not serious | ⨁◯◯◯ Very low |
| **CRT on vs CRT off** | | | | | | |
| All-cause mortality  640 patients  3 cohort studies | Serious^a^ | Not serious | Not serious | Serious^b^ | Not serious | ⨁◯◯◯ Very low |
| Ventricular arrythmias  640 patients  3 cohort studies | Serious^a^ | Not serious | Not serious | Serious^b^ | Not serious | ⨁◯◯◯ Very low |
| LVEDD  357 patients  2 observational studies | Serious^a^ | Not serious | Not serious | Serious^b^ | Not serious | ◯⨁◯◯ Low |
| **CRT vs no device/ICD** | | | | | | |
| All-cause mortality  1,911 patients  2 cohort studies | Serious^a^ | Not serious | Not serious | Not serious | Not serious | ◯⨁◯◯ Low |
| **CRT vs no device** | | | | | | |
| All-cause mortality  973 patients  2 cohort studies | Serious^a^ | Not serious | Not serious | Serious^b^ | Not serious | ⨁◯◯◯ Very low |
| **Biventricular pacing vs RV pacing** | | | | | | |
| Heart rate  67 patients  2 RCTs  1 observational study | Serious^a^ | Not serious | Not serious | Very serious^b^ | Not serious | ◯⨁◯◯ Low |
| RAP  44 patients  1 RCT  2 observational studies | Very serious^a^ | Not serious | Not serious | Very serious^b^ | Not serious | ⨁◯◯◯ Very low |
| MPAP  44 patients  1 RCT  2 observational studies | Very serious^a^ | Not serious | Not serious | Very serious^b^ | Not serious | ⨁◯◯◯ Very low |
| PCWP  44 patients  1 RCT  2 observational studies | Very serious^a^ | Not serious | Not serious | Very serious^b^ | Not serious | ⨁◯◯◯ Very low |
| TDCO  37 patients  1 RCT  1 observational study | Serious^a^ | Not serious | Not serious | Very serious^b^ | Not serious | ◯⨁◯◯ Low |
| PA saturation  37 patients  1 RCT  1 observational study | Serious^a^ | Not serious | Not serious | Very serious^b^ | Not serious | ◯⨁◯◯ Low |
| RVSWI  37 patients  1 RCT  1 observational study | Serious^a^ | Not serious | Not serious | Very serious^b^ | Not serious | ◯⨁◯◯ Low |
| **Biventricular pacing vs intrinsic rhythm** | | | | | | |
| Heart rate  30 patients  1 RCT  1 observational study | Serious^a^ | Not serious | Not serious | Very serious^b^ | Not serious | ◯⨁◯◯ Low |
| RAP  30 patients  1 RCT  1 observational study | Serious^a^ | Not serious | Not serious | Very serious^b^ | Not serious | ◯⨁◯◯ Low |
| MPAP  30 patients  1 RCT  1 observational study | Serious^a^ | Not serious | Not serious | Very serious^b^ | Not serious | ◯⨁◯◯ Low |
| PCWP  30 patients  1 RCT  1 observational study | Serious^a^ | Not serious | Not serious | Very serious^b^ | Not serious | ◯⨁◯◯ Low |
| TDCO  30 patients  1 RCT  1 observational study | Serious^a^ | Not serious | Not serious | Very serious^b^ | Not serious | ◯⨁◯◯ Low |
| PA saturation  30 patients  1 RCT  1 observational study | Serious^a^ | Not serious | Not serious | Very serious^b^ | Not serious | ◯⨁◯◯ Low |
| RVSWI  30 patients  1 RCT  1 observational study | Serious^a^ | Not serious | Not serious | Very serious^b^ | Not serious | ◯⨁◯◯ Low |

Supplementary Table 4. GRADE evidence profile

^a^Due to non-randomized study design and potential confounding

^b^Due to small number of studies and/or imprecise estimates

CRT, cardiac resynchronization therapy; HR, heart rate; ICD, implantable cardioverter-defibrillator; LVEDD, left ventricular end-diastolic diameter; MPAP, mean pulmonary artery pressure; PA, pulmonary artery; PCWP, pulmonary capillary wedge pressure; RAP, right atrial pressure; RCT, randomized controlled trial; RV, right ventricular; RVSWI, right ventricular stroke work index; TDCO, thermodilution cardiac output
